# The effects of different velocity loss thresholds on post-activation performance enhancement in basketball players

**DOI:** 10.7717/peerj.21550

**Published:** 2026-07-30

**Authors:** Han Wu, Ivan Jukic, Kai Xu, Patrick M. Holmberg, Bingze Du, Zhiming Shan, Wei Chen, Jiayin Jiang, Gengwu Guan, Qi Zhao

**Affiliations:** 1 Beijing Tieniu Sports Co., Ltd., Beijing, China; 2 Graduate School, Nanjing Sport Institute, Nanjing, Jiangsu, China; 3 Sport Performance Research Institute New Zealand (SPRINZ), Auckland University of Technology, Auckland, New Zealand; 4 Department of Health, Sport & Wellbeing, Faculty of Social and Applied Sciences, Abertay University, Dundee, United Kingdom; 5 School of Athletic Performance, Shanghai University of Sport, Shanghai, China; 6 School of Exercise and Nutrition Sciences, Queensland University of Technology, Brisbane, Queensland, Australia; 7 Physical Education College, Chengdu University, Chengdu, China; 8 School of Sports Training, Nanjing Sport Institute, Nanjing, Jiangsu, China; 9 Jiangsu Research Institute of Sports Science, Nanjing, Jiangsu, China; 10 Basketball Division, Jiangsu Training Center, Nanjing, Jiangsu, China

**Keywords:** Potentiation, Resistance training, Neuromuscular performance, Fatigue and potentiation, Velocity based training

## Abstract

**Aim:**

This study investigated the effects of different velocity loss (VL) thresholds during squat exercise at 80% of one-repetition maximum (1RM) on post-activation performance enhancement (PAPE) in basketball players.

**Methods:**

Nineteen male collegiate basketball players completed three familiarization sessions and four randomized crossover experimental sessions: control (passive rest), 5% VL, 10% VL, and 20% VL. Countermovement jump height (JH), peak power output (PPO), and reactive strength index modified (RSIm) were measured pre-test and at 3-min and 8-min post-squat. Linear mixed-effects models (LMMs) evaluated the effects of time, condition, and mean-centered relative strength on the outcome variables and repetition counts. To identify responders who exhibited meaningful improvements in jump height, the smallest worthwhile change (SWC) was calculated.

**Results:**

At the group level, no significant condition × time interactions or main effects of condition were observed for JH, PPO, or RSIm (*p* > 0.05), indicating no uniform PAPE effect across protocols. Furthermore, no statistically significant strength × time × condition interaction effects were observed. Repetition counts did not differ between the 5% and 10% VL conditions but were significantly lower in both conditions compared to the 20% VL condition (*p* < 0.01). Individual analysis showed the 20% VL condition yielded the highest number of responders (*n* = 11; 58%).

**Conclusion:**

Squat protocols using 5–20% VL at 80% 1RM did not produce significant group-level CMJ performance improvements, suggesting that a comprehensive warm-up may create a performance ceiling. Furthermore, these responses were not moderated by relative strength. However, the high inter-individual variability and responder rates at higher VL thresholds highlight the importance of individualized PAPE programming.

## Introduction

Basketball is a high-intensity team sport that requires athletes to repeatedly generate high power output (Wen et al., 2018), underpinning key technical actions such as rebounding (Berberet et al., 2025) and rapid changes of direction (Papla et al., 2022). Well-designed pre-game warm-up strategies can accelerate the attainment of match readiness (Cerrillo-Sanchis et al., 2024). The countermovement jump (CMJ) provides a rapid, practical indicator of lower-limb neuromuscular function and power in basketball players (Claudino et al., 2017), and is therefore widely used by strength and conditioning practitioners as a pre-match monitoring tool to gauge athletes’ current neuromuscular status.

Post-activation performance enhancement (PAPE) refers to the short-term improvement in physical performance that can occur following a conditioning activity (CA) involving maximal or near-maximal efforts (Blazevich & Babault, 2019; Boullosa, 2021; Boullosa et al., 2020; Brink, Constantinou & Torres, 2022a; Heynen et al., 2024; Hulet, Debeliso & Lawrence, 2025). Improvements in performance of ~2–10% have been reported, with optimal recovery periods typically ranging from 2.5 to 11 min (Xu et al., 2025). The magnitude of PAPE depends on a critical balance: maximizing neuromuscular enhancement while minimizing the acute fatigue induced by the CA. This balance is challenging to strike due to high inter-individual variability (Krzysztofik et al., 2021a, 2021b), making the “dosing” of the CA a central challenge for practitioners.

Velocity-based training (VBT) offers a potential solution to this dosing problem by using barbell velocity to quantify training effort and fatigue in real time. Velocity loss (VL) serves as a reliable metric to monitor and control fatigue during resistance training, often by terminating a set once a specific VL is reached. VL thresholds correlate strongly with both the percentage of repetitions completed relative to a maximum (Weakley et al., 2021) and the accumulation of fatigue-related metabolites like lactate and ammonia (Sánchez-Medina & González-Badillo, 2011). A recent systematic review suggested that greater VL is generally associated with less favourable acute performance, and that heavier loads (≥80% 1RM) may partially attenuate this effect (Jukic et al., 2023a). While acute evidence in that review could not be pooled quantitatively, descriptive trend analyses supported this pattern, further noting that lower VL thresholds (≤15%) appeared to be most conducive to longer-term improvements in sprint performance and CMJ height. Consistent with the practical demands of basketball, a study in female basketball players reported that squatting at 80% 1RM elicited greater improvements in jump and change-of-direction performance than other loading conditions. Therefore, utilizing low VL thresholds during heavy-load (≥80% 1RM) conditioning activities may optimize the fatigue-enhancement balance necessary to elicit PAPE in powerful athletes like basketball players.

However, the literature remains equivocal on the optimal VL threshold and load for inducing PAPE. For instance, Tsoukos et al. (2019) found that bench press exercises at 60% 1RM with a 10% VL yielded the greatest acute improvements. In contrast, Collins et al. (2024) reported no improvement in ballistic push-up performance at a 10% VL. More specific to this study, Yuan et al. (2023) investigated squats at 85% 1RM with two sets and reported that a 5% VL threshold was optimal for PAPE in track and field athletes. Meanwhile, a recent meta-analysis suggested that a single-set CA performed with VL ≤10% and load ≥80% 1RM may be associated with larger PAPE effects (Fan et al., 2025). Collectively, these mixed findings—likely influenced by exercise modality and participant characteristics—underscore ongoing uncertainty.

This uncertainty is compounded by a more fundamental methodological concern: the warm-up protocol. Recent evidence suggests that PAPE may not provide any additional benefit when implemented after a truly comprehensive warm-up (Xu et al., 2025). An insufficient warm-up can, therefore, lead to an overestimation of the PAPE effect, suggesting that many published findings might simply represent a “catch-up” warm-up effect rather than a true PAPE phenomenon (Bishop, 2003; Xu et al., 2025). These conflicting results may be further exacerbated by other methodological limitations in existing PAPE research, such as inadequate blinding (Blazevich & Babault, 2019) and feed-forward effects during testing (Brink, Constantinou & Torres, 2022b). Therefore, accurately assessing the true effects of PAPE requires rigorous methodological controls, particularly a comprehensive and standardized warm-up.

Accordingly, the purposes of this study were twofold: (1) to investigate the optimal VL threshold for inducing PAPE during squat exercises, and (2) to determine whether PAPE provides any additional performance enhancement beyond that achieved by an adequate warm-up alone. We hypothesized that (1) among the tested VL thresholds, 5% VL would elicit the most favorable PAPE response, but (2) no CA protocol would provide a statistically significant performance enhancement over the comprehensive raise, activate, mobilize, and potentiate (RAMP) warm-up alone.

## Materials and Methods

### Experimental approach to the problem

This study utilized a single-blind, randomized, repeated-measures crossover design. The order of the four experimental conditions was randomized for each participant before the experimental sessions using the RAND function in Microsoft Excel (Microsoft Corporation, Redmond, WA, USA). Participants were informed that the study aimed to compare different “recovery strategies” for explosive performance but were kept blind to the specific conditioning protocols (*i.e*., the VL thresholds). Data analysts were not blinded to the experimental conditions because condition labels were required for the repeated-measures analysis and interpretation of condition-specific effects. All protocols were investigator-controlled, and participants were fully debriefed on the true purpose of the study following the completion of all sessions.

**Figure 1:**
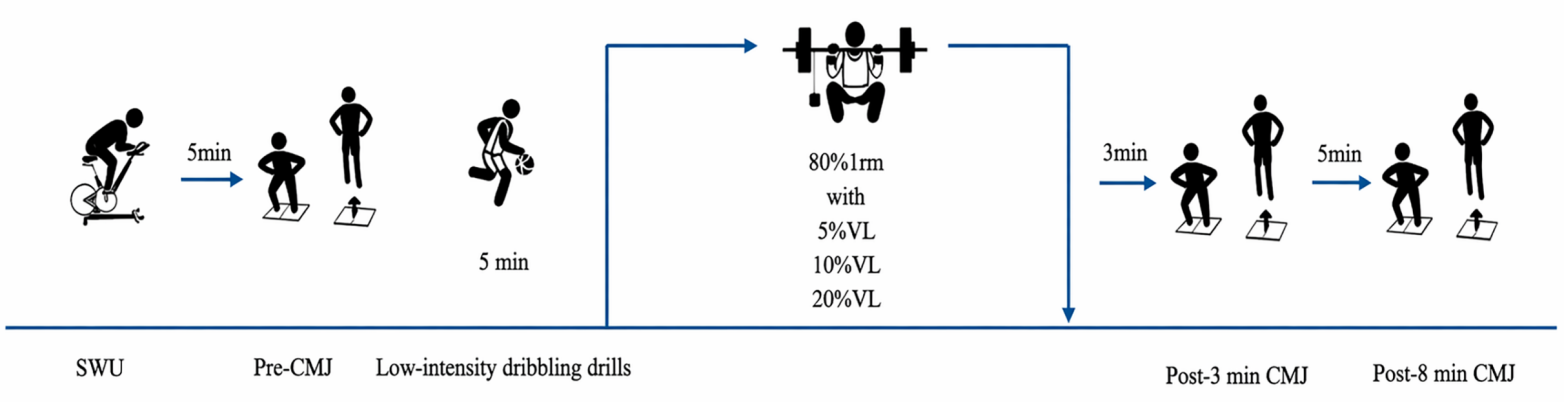
PAPE procedure flowchart. Participants completed a standardized warm-up (SWU), baseline countermovement jump (Pre-CMJ), and low-intensity dribbling drills before performing the conditioning activity at 80% 1RM under four conditions (Control, 5%VL, 10%VL, and 20%VL). CMJ performance was reassessed at 3 min and 8 min post-intervention. CMJ, countermovement jump; 1RM, one-repetition maximum; VL, velocity loss.

Each participant completed seven laboratory visits, separated by at least 48 h, and all sessions were conducted at the same time of day (16:00 ± 2:00 h). The first visit involved baseline measurements, including anthropometrics (height, body mass, body composition) and determination of the back squat one-repetition maximum (1RM). During the second and third sessions, participants practiced the full experimental protocol, including performing squats with velocity feedback (at 80% 1RM) and practicing the countermovement jump (CMJ) on a force plate to minimize learning effects. During the four experimental sessions, participants first completed a standardized warm-up (Table 1). Following this, they performed one of four squat protocols in a randomized order: CA using 80% 1RM terminated at 5% VL, 10% VL, or 20% VL, or a control condition (no CA). After the assigned protocol and a passive recovery, jump performance was assessed *via* CMJ tests at 3 min and 8 min post-protocol. The primary outcome measures derived from the CMJ were jump height (JH), peak power output (PPO), and the modified reactive strength index (RSIm). The complete experimental timeline is illustrated in Fig. 1.

**Table 1:** Warm up protocol.

	Description	Volume
High knees	Walk while lifting the knee to the chest, rising onto the toes of the opposite leg.	Four sets per leg, 20 m per set
Leg swings	Actively swing the leg forward into hip flexion, keeping the knee extended and ankle plantarflexed.	Four sets per leg, 20 m per set
Hurdler’s walk	Flex the hip (~90°), abduct, and externally rotate the knee as if stepping over a hurdle.	Four sets per leg, 20 m per set
Butt kicks	Rapidly kick heels toward the buttocks while moving forward.	Four sets per leg, 20 m per set
Tip-Toe walk	Walk forward while alternating plantarflexion (rising onto toes) with each step.	Four sets per leg, 20 m per set

### Participants

Nineteen male basketball players from Nanjing Sport Institute (mean ± SD: age = 18.68 ± 0.84 years; height = 184.74 ± 6.11 cm; body mass = 77.50 ± 10.57 kg; body fat percentage = 13.11 ± 10.23%) voluntarily participated in this study. An *a priori* power analysis was conducted in G*Power (version 3.1.9; Universität Düsseldorf, Germany) for a repeated-measures ANOVA using a medium effect size (Cohen’s f = 0.25), an assumed correlation among repeated measures of 0.85 (Xu et al., 2025), α = 0.05, and 80% power, which indicated a minimum required sample size of 12 participants. To account for potential attrition due to injury, competition, or other factors, seven additional participants were recruited.

Inclusion criteria required participants to: (1) be competing in basketball; (2) have a minimum of 2 years of structured resistance training experience; and (3) hold a second-class athlete certification or higher. Based on these criteria and the classification proposed by McKay et al. (2021), all participants were classified as trained individuals (Level II). Exclusion criteria included any lower-limb musculoskeletal injury or lower back pain within the previous 6 months that could impair participation. Participants were instructed to avoid high-intensity exercise, caffeine, alcohol, and other stimulants for 24 h prior to testing. The study was conducted in accordance with the Declaration of Helsinki and was approved by the Ethics Committee of Nanjing Sport Institute (approval number: RT-2015-10). All participants provided written informed consent prior to participation and were informed that the study used a blinded design, meaning they would not be told their assigned condition.

### Familiarisation sessions and 1RM test

During the first visit, participants’ 1RM in the parallel back squat was determined. The session began with a warm-up consisting of 5 min of stationary cycling and a series of dynamic lower-limb stretches (detailed in Table 1). For the test itself, a linear position transducer (GymAware, Kinetic Performance, Canberra, Australia) was attached to the barbell to monitor mean concentric velocity (MV) in real time (Orange et al., 2020). Squat depth was standardized using a 50 cm box positioned inside the squat rack. Participants were instructed to perform a parallel back squat, making light contact with the box before ascending. A trained observer verified a parallel thigh position at box contact and used yoga blocks to adjust the height as needed.

Testing began with a 40 kg load, and initial increments ranged from 10 to 20 kg. A strength and conditioning coach determined subsequent load increments based on the MV of the previous lift. Rest intervals were prescribed based on velocity: 5 min of rest was provided when MV was <0.5 m/s, 3 min when 0.5 ≤ MV ≤ 0.7 m/s, and 1 min when MV was >0.7 m/s (Sánchez-Medina et al., 2017). As participants approached their maximum, increments were reduced to 0.5–5 kg. The test was terminated when the participant failed to complete a lift, and the highest successfully lifted load was recorded as the 1RM.

The second and third sessions were used for participant familiarization and to establish a reliable baseline MV at 80% 1RM, which was used to monitor strength throughout the experimental period. After completing the same warm-up, participants performed one set each at 60% 1RM (three repetitions), 70% 1RM (two repetitions), and 80% 1RM (one repetition), with 3, 4, and 5 min of rest (Sánchez-Medina et al., 2017), respectively. Following this, they performed three sets of three maximal-effort repetitions at 80% 1RM. During these efforts, the eccentric phase was self-paced, and the concentric phase was performed with maximal effort. Verbal feedback on MV was provided after each repetition. From each of these three sets, the single highest MV was identified. The average of the two highest MV values (out of the three sets) was calculated and recorded as the participant’s baseline MV. During the subsequent experimental interventions, if a participant’s warm-up MV at 80% 1RM deviated from this baseline by >0.06 m/s (Banyard, Nosaka & Haff, 2017; Weakley et al., 2021), the load was adjusted (by ±5% of 1RM per 0.06 m/s deviation) to ensure the MV was within the target range before the PAPE protocol began.

### Experimental sessions

Upon arrival for each of the four experimental sessions, participants drew a sealed envelope indicating the conditioning activity (CA) for that day. The researcher opened the envelope, but the participant remained blind to the specific protocol. Each session followed the same timeline: a standardized warm-up, baseline CMJ assessment, the assigned intervention protocol, and post-intervention CMJ assessments.

The session began with a general warm-up of 5 min of stationary cycling and dynamic stretching (Table 1). Participants then performed three submaximal (~80% effort) CMJs for readiness, followed by 5 min of passive rest. Baseline CMJ performance was then assessed (details below). Following the baseline assessment, all participants performed 5 min of low-intensity dribbling (*e.g*., stationary dribbling, dribbling while moving; no running) to maintain body temperature. The protocol then differed based on the assigned condition.

PAPE Conditions (5% VL, 10% VL, 20% VL): Participants first completed a squat-specific warm-up identical to the familiarization sessions (sets at 60%, 70%, and 80% 1RM). Immediately after, they performed the CA, which consisted of maximal-effort squats at 80% 1RM. A linear position transducer (GymAware) was preset to the assigned VL threshold (5%, 10%, or 20%). Real-time velocity feedback was provided verbally after each repetition, and the set was terminated once the prescribed VL was reached. The number of repetitions and actual VL were recorded. Control Condition: Following the 5-min dribbling period, participants proceeded directly into a passive rest period.

In all four conditions, CMJ performance was reassessed at 3 min and 8 min after the completion of the protocol (or equivalent rest period). CMJ were performed on two portable force plates (Kun Wei FP-6036, Kunwei Technology Co., Ltd., Shanghai, China) sampling at 1,000 Hz. The reliability and validity of this system have been established previously (Mao et al., 2024). Data were collected using the force platform’s accompanying software (GuanNeng Force Measurement System, Guangzhou, China). During each jump, the participants stood on the force plate in an upright position with their hands placed on their hips to minimize the influence of arm swing on JH. Participants rapidly descended to a self-selected squat depth and immediately executed maximal-effort vertical jumps. The participants were instructed to “jump as high as possible” and were verbally encouraged throughout testing. After each jump, the force–time curve was visually inspected to verify data validity; trials in which the system failed to accurately detect the take-off instant were excluded.

- JH was calculated from take-off velocity: JH = Vtake-off²/2 g, (*where Vtake-off is the take-off velocity and g is the acceleration due to gravity* (Eythorsdottir et al., 2024)).
- PPO was calculated as: PPO = F × V (*where F is force and V is velocity; PPO was defined as the highest instantaneous power value observed during the concentric phase of the CMJ* (Owen et al., 2014))
- The RSIm was calculated as: RSIm = JH/TToff (*where TToff is the time to take-off* (McMahon, Jones & Comfort, 2022))

To assess individual responses, the smallest worthwhile change (SWC) was calculated as 0.2 × the baseline standard deviation (Godwin, Dhone & Newman, 2023). An individual was classified as a “responder” if their post-intervention JH improvement exceeded this SWC threshold.

### Statistical analysis

All data are reported as mean ± standard deviation (SD) unless otherwise specified. Statistical analyses were performed using SPSS 22.0 (IBM Corp., Armonk, NY, USA) and R (version 4.5.0, R Core Team, 2025).

The reliability of CMJ measurements was assessed using the intraclass correlation coefficient (ICC), coefficient of variation (CV), and standard error of measurement (SEM). ICC values were interpreted as follows: poor reliability (<0.5), moderate reliability (0.5–0.75), good reliability (0.75–0.90), and excellent reliability (>0.90) (Koo & Li, 2016). The CV was derived from log-transformed data to account for proportional error, calculated as: 100(e^SDlog^/100−1). Values defined as <10% were considered reliable (Turner et al., 2015).

The primary analysis used linear mixed-effects models (LMMs) to assess the effects of condition and time on each outcome variable (JH, PPO, and RSIm). Models were fitted using restricted maximum likelihood (REML) with the lme4 package (Bates et al., 2015). Fixed effects included condition (four levels), time (three levels: baseline, 3-min, 8-min), and their interaction (condition × time). To evaluate whether strength level moderated the PAPE response, mean-centered relative strength (1RM normalized to body mass) was included as a continuous covariate (Seitz & Haff, 2016), along with the condition × time × strength interaction term. Participants were modelled with random intercepts to account for inter-individual variability. Model assumptions were verified using Q–Q plots, Shapiro–Wilk tests, and DHARMa package diagnostics. Significant fixed effects or interactions were followed up with Tukey’s *post hoc* tests. A separate LMM was used to analyze the number of repetitions completed, with condition as a fixed effect and participant as a random intercept.

## Results

### Reliability analysis

Baseline CMJ measurements demonstrated good to excellent reliability across all variables. JH (ICC = 0.94 [0.91–0.96]; CV = 4.52%), PPO (ICC = 0.97 [0.95–0.98]; CV = 2.99%), and RSIm (ICC = 0.84 [0.78–0.89]; CV = 8.53%) all showed good-to-excellent ICCs and acceptable CVs. Specific SEM and SWC values are presented in Table 2.

**Table 2:** Results of the reliability analysis.

Indicator	ICC	CV	SEM	SWC
JH	0.940	4.52%	1.20 cm	1.16 cm
PPO	0.970	2.99%	104.5 W	122.76 W
RSIm	0.841	8.53%	0.037	0.019

**Note:**

ICC, Intraclass Correlation Coefficient; CV, Coefficient of Variation; SEM, Standard Error of Measurement; SWC, Smallest Worthwhile Change; CMJ, Countermovement Jump; RSIm, Reactive Strength Index (Modified); W, Watt.

### Jump performance outcomes

For the linear mixed-effects models (LMMs), residual diagnostics indicated slight deviations from normality. However, simulated residual analysis (DHARMa) suggested adequate model fit. Furthermore, parametric bootstrap results (1,000 resamples) were consistent with the original statistical estimates, confirming the robustness of the findings.

The LMM revealed no statistically significant (condition × time × strength) interaction effects for JH (*p* = 0.98), PPO (*p* = 0.82), or RSIm (*p* = 0.67). In addition, no significant condition × time interactions were observed for any outcome (all *p* > 0.05), and strength did not moderate the time-dependent responses (time × strength, all *p* > 0.05).

**Figure 2:**
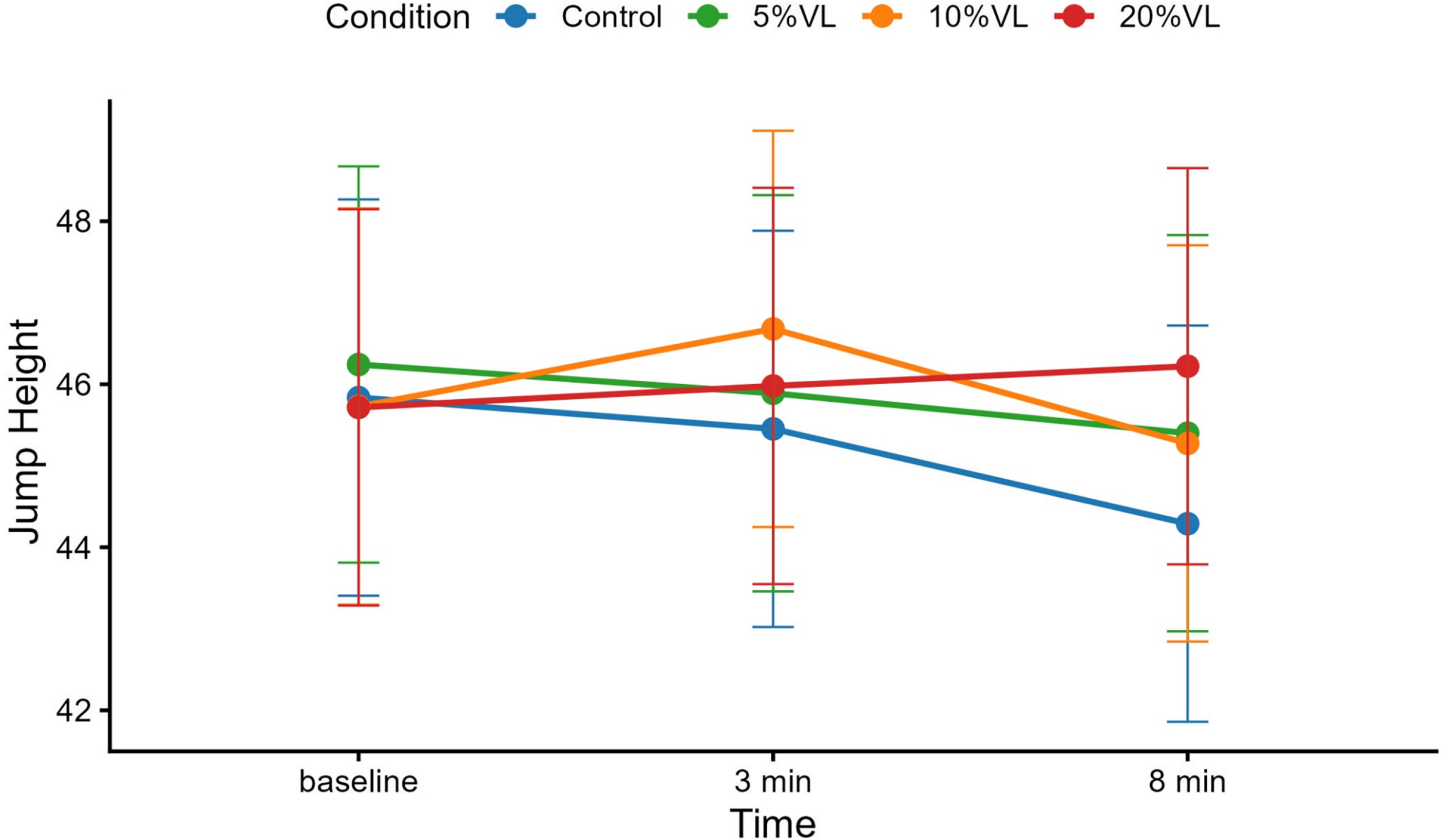
Estimated marginal means of CMJ jump height across conditions and time points. Estimated marginal means with 95% confidence intervals at baseline, 3 min, and 8 min for Control, 5%VL, 10%VL, and 20%VL. Points represent model-estimated means, and error bars indicate 95% confidence intervals. CMJ, countermovement jump; VL, velocity loss.

**Figure 3:**
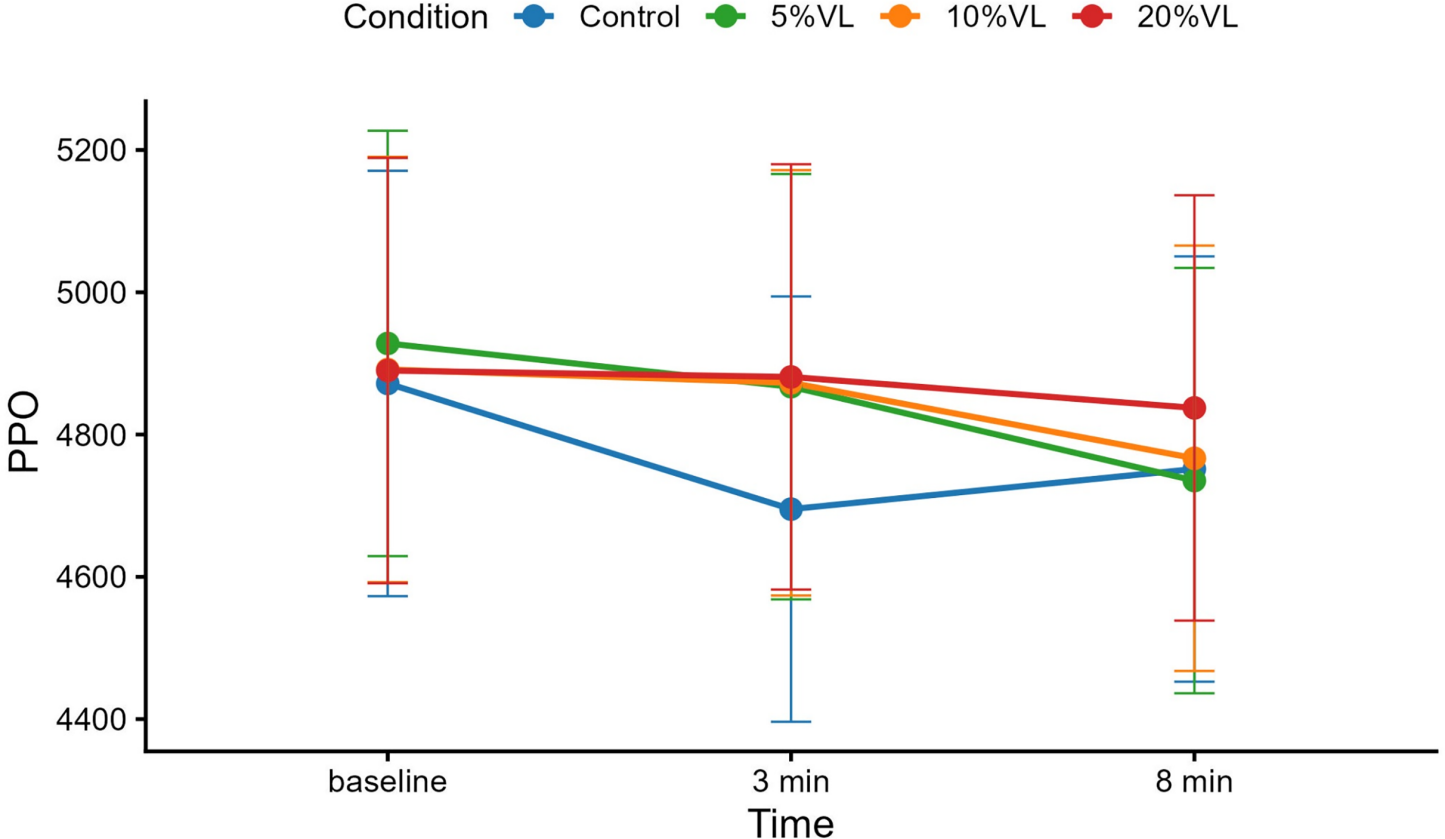
Estimated marginal means of peak power output (PPO) across conditions and time points. Estimated marginal means with 95% confidence intervals at baseline, 3 min, and 8 min for Control, 5%VL, 10%VL, and 20%VL. Points represent model-estimated means, and error bars indicate 95% confidence intervals. PPO, peak power output; VL, velocity loss.

**Figure 4:**
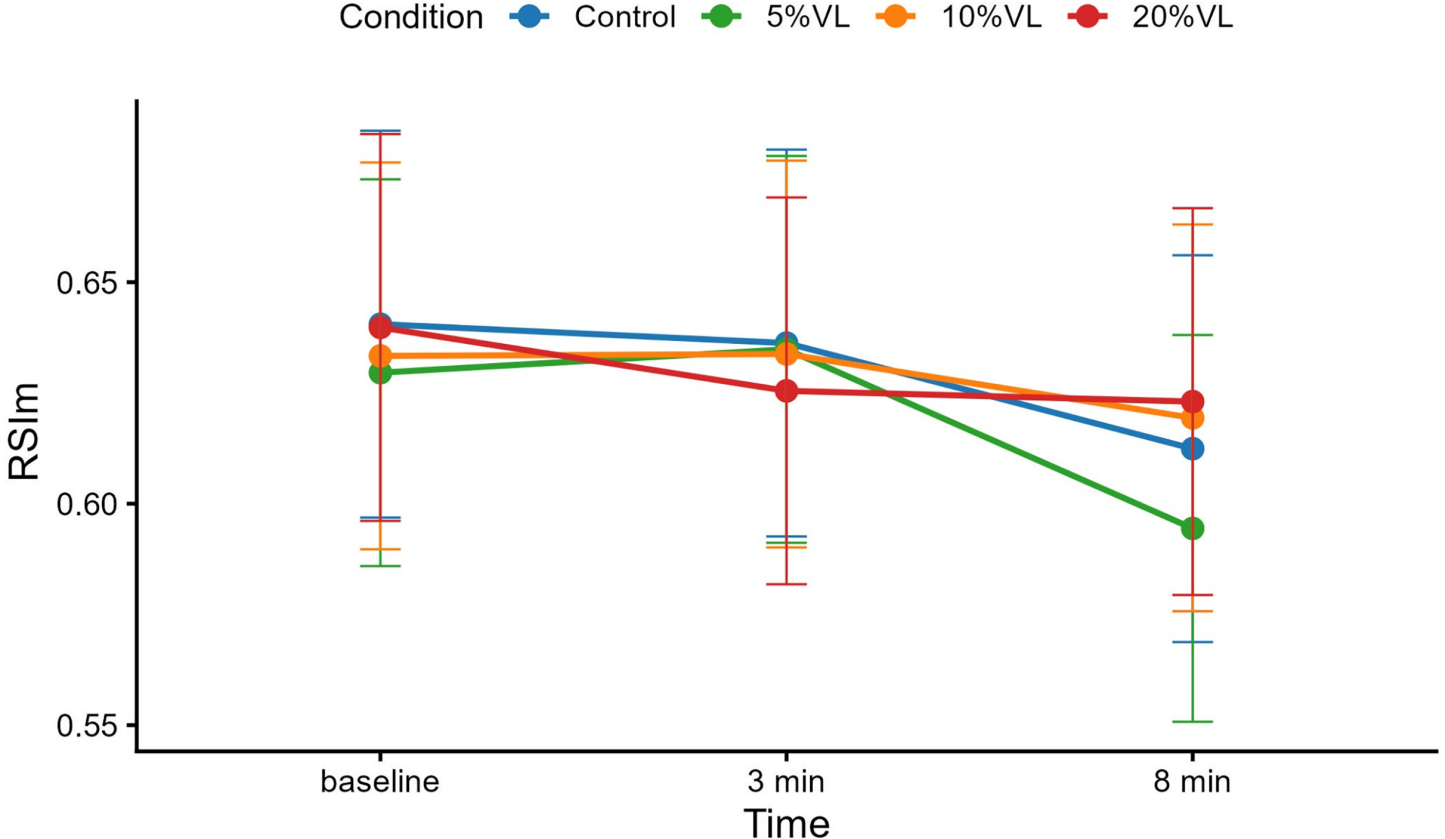
Estimated marginal means of RSIm across conditions and time points. Estimated marginal means with 95% confidence intervals at baseline, 3 min, and 8 min for Control, 5%VL, 10%VL, and 20%VL. Points represent model-estimated means, and error bars indicate 95% confidence intervals. RSIm, reactive strength index modified; VL, velocity loss.

For main effects, significant strength effects were observed for JH and RSIm, whereas significant time effects were observed for PPO and RSIm. *Post hoc* analyses indicated that baseline values were significantly higher than those at 8 min post exercise (PPO: Estimate = 122.6, *p* = 0.01, d = 0.46; RSIm: Estimate = 0.02, *p* = 0.03, d = 0.41). As illustrated in Figs. 2–4, the descriptive statistics presented in Table 3 and the detailed statistical results provided in File S1, group-level jump performance remained largely unchanged from baseline at both the 3-min and 8-min post-intervention time points, regardless of the CA or control protocol.

**Table 3:** Descriptive statistics of various variables across different groups and time points.

Indicator	Condition	Baseline	Post-3 min	Post-8 min
**JH**	Control	45.84 ± 5.40	45.45 ± 5.01	44.30 ± 5.48
	VL5	46.24 ± 2.70	45.89 ± 4.30	45.40 ± 3.98
	VL10	45.13 ± 5.17	46.68 ± 5.62	45.27 ± 5.70
	VL20	45.72 ± 5.14	45.98 ± 5.87	46.22 ± 6.12
**PPO**	Control	4,871.79 ± 625.50	4,695.16 ± 703.37	4,751.5 ± 542.49
	VL5	4,928.00 ± 659.30	4,867.20 ± 598.80	4,735.20 ± 659.19
	VL10	4,891.40 ± 643.40	4,872.70 ± 596.38	4,766.63 ± 567.34
	VL20	4,889.90 ± 645.17	4,881.00 ± 687.37	4,837.42 ± 740.12
**RSIm**	Control	0.64 ± 0.10	0.64 ± 0.09	0.61 ± 0.11
	VL5	0.63 ± 0.09	0.64 ± 0.08	0.60 ± 0.08
	VL10	0.63 ± 0.09	0.63 ± 0.07	0.62 ± 0.11
	VL20	0.64 ± 0.10	0.63 ± 0.10	0.62 ± 0.11

**Notes:**

VL5, VL10, and VL20 denote the 5%, 10%, and 20% velocity-loss conditions, respectively.

JH, jump height; PPO, peak power output; RSIm, reactive strength index modified. Values are mean ± SD.

**Figure 5:**
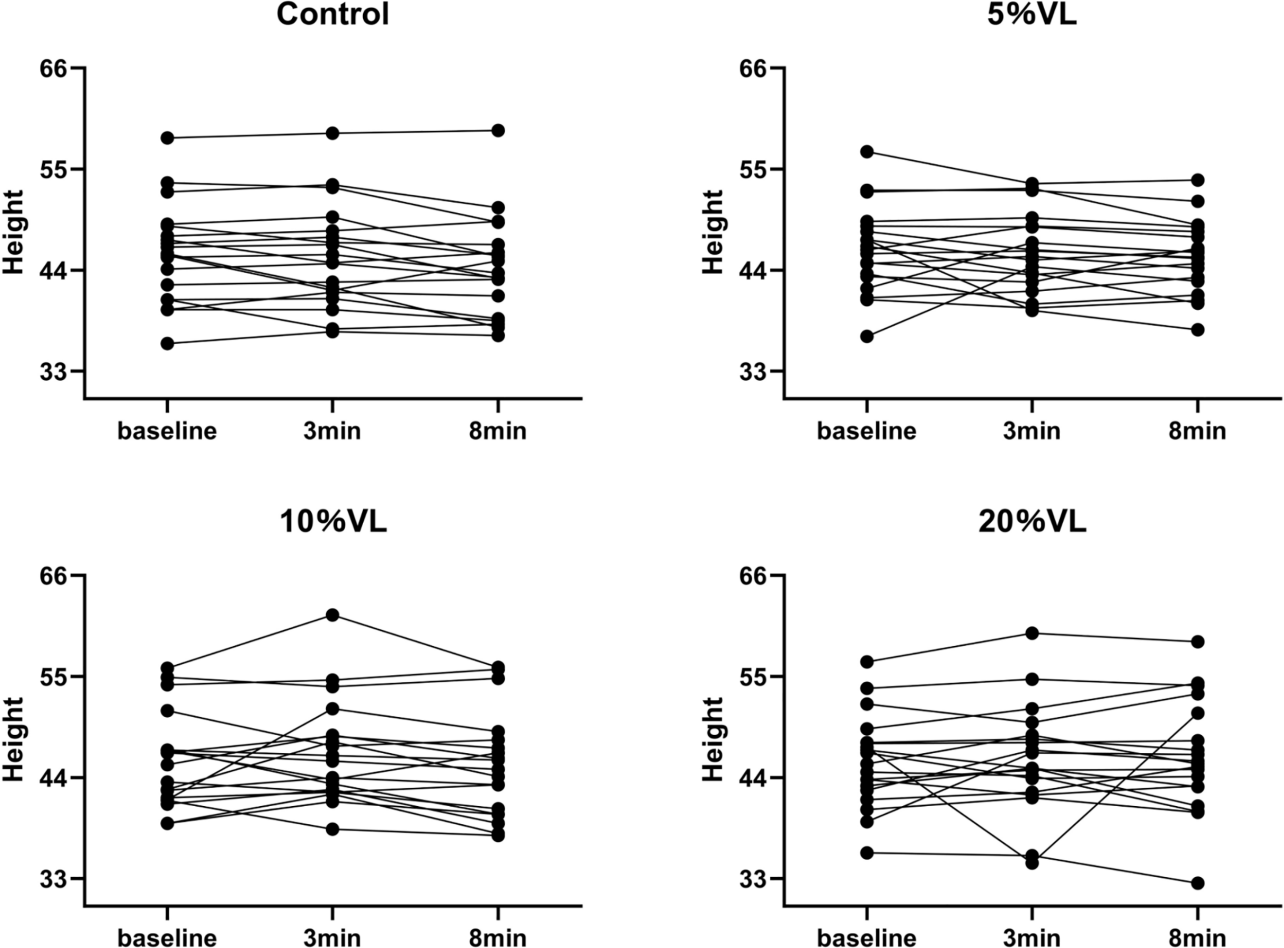
Individual changes in jump height from baseline to post-intervention across conditions. Each line represents one participant’s CMJ jump height measured at baseline, 3 min, and 8 min. Panels show Control, 5%VL, 10%VL, and 20%VL conditions. CMJ, countermovement jump; VL, velocity loss.

Despite the lack of significant group-level effects, there was considerable inter-individual variability in the response to the protocols, as shown in Fig. 5. A total of 29 “responder” instances (*i.e*., improvements in JH exceeding the SWC) were observed across the 76 total interventions (19 participants × four conditions). These responses were distributed across all conditions: 11 responders were identified in the 20% VL group, 10 in the 10% VL group, and 5 in the 5% VL group. Notably, three participants were also classified as responders in the control condition. The largest individual improvement observed was 24% (9.9 cm).

As expected, the number of repetitions completed differed significantly between the CA conditions (*p* < 0.001). *Post hoc* tests (detailed in Table 4) showed that the 20% VL group performed significantly more repetitions than both the 10% VL (*p* < 0.01) and 5% VL (*p* < 0.01) groups. No significant difference in repetition count was found between the 5% and 10% VL groups (*p* = 0.108).

**Table 4:** Results of repetitions and velocity loss comparison across different VL groups.

Group	Repetition	Velocity loss
5%	4.21 ± 1.27	8.95 ± 3.27
10%	5.47 ± 2.41	15.24 ± 3.76
20%	9.42 ± 2.49*	27.46 ± 4.80

**Note:**

* Indicates a significant difference between the 20% group and the 5% and 10% groups, *p* < 0.01.

## Discussion

This study investigated the acute effects of different VL thresholds (5%, 10%, 20%) during a back squat CA on subsequent CMJ performance. The primary finding was that no VL threshold produced a significant group-level improvement in JH, PPO, or RSIm at either 3 or 8 min post-CA. However, this null group finding masked considerable inter-individual variability; 11 “responders” were identified in the 20% VL group, 10 in the 10% VL group, and 5 in the 5% VL group, with one individual improving by 24% (9.9 cm).

A primary objective of this study was to determine if PAPE could enhance performance beyond a comprehensive warm-up. The lack of any group-level improvements suggests it may not. Our protocol, which followed the RAMP principle (Table 1), likely elevated baseline performance to a ceiling, leaving no room for a subsequent CA to produce further potentiation enhancement; notably, both PPO and RSIm were significantly lower at 8 min than at baseline. This finding supports recent work by Xu et al. (2025) who found that PAPE provided no additional benefit after a thorough, sport-specific warm-up. This “warm-up effect” may explain many positive findings in the literature; an insufficient warm-up can lead to an overestimation of PAPE, where the “effect” is simply the participant completing their warm-up (Bishop, 2003). Our results are also consistent with Rappelt et al. (2024) and Lucas et al. (2025), who both suggested that an intense warm-up may obscure any subsequent PAPE effects. Despite these null group-level effects, considerable inter-individual variability was evident; however, when mean-centered relative strength was included as a continuous covariate in the LMM to test for moderation, no strength-related interaction effects were detected, suggesting that baseline strength did not meaningfully influence the time- or condition-dependent responses in this cohort. This contrasts with literature suggesting stronger individuals may potentiate more (Wilson et al., 2013; Yang et al., 2024). Imperfect VL control, together with high variability, may have reduced our ability to detect clear strength-related effects. Collectively, these findings reinforce that “responder” status is multifactorial and highlight the need for individualized potentiation strategies.

The second major finding was that VL thresholds appeared to be an unreliable method for regulating the PAPE stimulus. A core assumption of this study’s design was that 5%, 10%, and 20% VL would create three distinct and progressive stimuli. However, this theoretical progression was not reflected in the data. There was no significant difference in the number of repetitions completed between the 5% and 10% VL groups. More tellingly, we observed significant protocol crossover: six participants in the 5% VL group exceeded 10% VL, and three in the 10% group exceeded 20% VL. This aligns with findings that the VL–repetition relationship is weaker at lower VLs (Jukic et al., 2023a, 2023b) and can be inconsistent (Jukic et al., 2023c). Given that this relationship may also be influenced by inter-individual and day-to-day factors (*e.g*., sex, training history, and affect/arousal stability), practitioners should interpret and apply VL-based set termination with caution (Jukic et al., 2023b, 2023c). While VL is a valid tool for monitoring fatigue, our results show it is a poor tool for dosing a PAPE stimulus, as it failed to create a consistent training volume.

The high inter-individual variability seen in our data (Fig. 5) suggests that PAPE is not a uniform phenomenon. Our finding that approximately half the participants responded in the 10% VL and 20% VL groups mirrors the “responders *vs*. non-responders” observations of Krzysztofik et al. (2021a) and Godwin, Dhone & Newman (2023). This variability, which even saw three participants respond in the control condition, suggests that a “one-size-fits-all” PAPE protocol is ineffective. Factors like fiber type, training history, and acute fatigue levels can significantly alter the optimal fatigue-potentiation balance (Blazevich & Babault, 2019; Boullosa, 2021; Wilson et al., 2013). This highlights the need for practitioners to individualize potentiation strategies rather than applying a universal prescription.

This study has several limitations worthy of consideration while interpreting the findings. First, the design was single-blind, which may have introduced bias. Second, an individualized load-velocity profile was not established for each participant. While our standardized velocity adjustment (0.06 m/s ≈ 5% 1RM) was derived from established literature, future research should utilize individualized profiles for greater precision. Third, testing barbell velocity at 80% 1RM may be somewhat redundant, as this value can be obtained during the 1RM assessment by calculating the load–velocity profile. Fourth, our findings are limited to male basketball players and may not generalize to other populations. Moreover, our post-test measurements were conducted at only two time points (3 and 8 min), which may not have captured the optimal PAPE window for every individual (Wilson et al., 2013). Fifth, PAPE may depend on the type of CA; for CMJ, lower-force, higher-velocity stimuli may be more effective than heavy-load squatting (Ritchie et al., 2024). Additionally, the repeated testing itself may have acted as a potentiating or fatiguing confound (Brink, Constantinou & Torres, 2022b; Cuenca-Fernández et al., 2024). Lastly, our reliance on JH, PPO, and RSIm may have masked subtle changes in performance, as these metrics are heavily influenced by jump strategy (Jidovtseff et al., 2014; Pérez-Castilla et al., 2021; Watkins et al., 2023) and may not fully capture underlying changes in neuromuscular output.

## Conclusion

The results of the study demonstrate that squatting at 5%, 10%, or 20% VL thresholds did not enhance group-level CMJ performance in trained basketball players who had already completed a comprehensive RAMP warm-up. This suggests that a thorough warm-up may create a performance ceiling, rendering additional PAPE protocols ineffective. Furthermore, VL was found to be an unreliable tool for controlling the CA stimulus, with significant crossover between groups and no difference in repetitions between the 5% and 10% conditions. Given the high individual variability, coaches should prioritize the quality of the warm-up and rely on their professional experience and understanding of individual athlete characteristics to decide whether additional potentiation activities are necessary, rather than depending on a single velocity-loss prescription.

## Supplemental Information

10.7717/peerj.21550/supp-1 Supplemental Information 1 CMJ dataset for statistical analyses (jump height, PPO, RSIm). The analysis dataset contained repeated CMJ measurements collected at three time points (baseline, 3 min post, and 8 min post) under four conditions (Control, 5%VL, 10%VL, and 20%VL). Each row represented one observation for one participant at a given condition and time point. The dataset included a de-identified participant identifier, condition, time, and the dependent variables derived from the force-platform CMJ trials: jump height (cm), peak power output (PPO) (W), and reactive strength index modified (RSIm) (dimensionless; calculated as jump height divided by time-to-takeoff). If multiple CMJ trials were performed at each time point, the best trial (highest jump height) was used for analysis

10.7717/peerj.21550/supp-2 Supplemental Information 2 Codebook.

10.7717/peerj.21550/supp-3 Supplemental Information 3 Detailed linear mixed-model results for JH, PPO, and RSIm. The detailed Type III ANOVA results from the linear mixed models for jump height (JH), peak power output (PPO), and modified reactive strength index (RSIm). Fixed effects included time, condition, relative strength, and all interaction terms. The models were fitted using restricted maximum likelihood, and p values were derived using Type III tests with Kenward–Roger degrees of freedom.

## Additional Information and Declarations

### Data Availability

The following information was supplied regarding data availability:

The raw data is available in the Supplemental Files.
