# Supplementary material for: The effects of different velocity loss thresholds on post-activation performance enhancement in basketball players
Source: PeerJ. 2026 Jul 30;14:e21550. doi: 10.7717/peerj.21550 (PMC13429104; doi:10.7717/peerj.21550)
Supplement: Supplemental Information 2 [file peerj-14-21550-s002.docx]

1.The Name column: each number represents one participant.
2.The Group column: 20 indicates the 20% velocity-loss group, 10 indicates the 10% velocity-loss group, 5 indicates the 5% velocity-loss group, and 0 indicates the quiet control group.
