# Supplementary material for: The effects of different velocity loss thresholds on post-activation performance enhancement in basketball players
Source: PeerJ. 2026 Jul 30;14:e21550. doi: 10.7717/peerj.21550 (PMC13429104; doi:10.7717/peerj.21550)
Supplement: Supplemental Information 3 — The detailed Type III ANOVA results from the linear mixed models for jump height (JH), peak power output (PPO), and modified reactive strength index (RSIm). Fixed effects included time, condition, relative strength, and all interaction terms. The models were fitted using restricted maximum likelihood, and p values were derived using Type III tests with Kenward–Roger degrees of freedom. [file peerj-14-21550-s003.docx]

## JH

Table 1. Type III ANOVA for JH

| **Effect** | **Sum Sq** | **Mean Sq** | **NumDF** | **DenDF** | **F value** | **Pr(>F)** |
| --- | --- | --- | --- | --- | --- | --- |
| time | 21.563 | 10.782 | 2.000 | 187.000 | 1.651 | 0.195 |
| condition | 22.023 | 7.341 | 3.000 | 187.000 | 1.124 | 0.341 |
| strength | 80.185 | 80.185 | 1.000 | 17.000 | 12.279 | 0.003 |
| time:condition | 31.879 | 5.313 | 6.000 | 187.000 | 0.814 | 0.561 |
| time:strength | 1.568 | 0.784 | 2.000 | 187.000 | 0.120 | 0.887 |
| condition:strength | 47.000 | 15.667 | 3.000 | 187.000 | 2.399 | 0.069 |
| time:condition:strength | 6.738 | 1.123 | 6.000 | 187.000 | 0.172 | 0.984 |

## PPO

Table 2. Type III ANOVA for PPO

| **Effect** | **Sum Sq** | **Mean Sq** | **NumDF** | **DenDF** | **F value** | **Pr(>F)** |
| --- | --- | --- | --- | --- | --- | --- |
| time | 572,345.377 | 286,172.689 | 2.000 | 187.000 | 4.096 | 0.018 |
| condition | 294,777.572 | 98,259.191 | 3.000 | 187.000 | 1.406 | 0.242 |
| strength | 1,043.533 | 1,043.533 | 1.000 | 17.000 | 0.015 | 0.904 |
| time:condition | 308,269.886 | 51,378.314 | 6.000 | 187.000 | 0.735 | 0.622 |
| time:strength | 17,538.538 | 8,769.269 | 2.000 | 187.000 | 0.126 | 0.882 |
| condition:strength | 382,284.295 | 127,428.098 | 3.000 | 187.000 | 1.824 | 0.144 |
| time:condition:strength | 205,216.073 | 34,202.679 | 6.000 | 187.000 | 0.490 | 0.816 |

## RSIm

Table 3. Type III ANOVA for RSIm

| **Effect** | **Sum Sq** | **Mean Sq** | **NumDF** | **DenDF** | **F value** | **Pr(>F)** |
| --- | --- | --- | --- | --- | --- | --- |
| time | 0.025 | 0.012 | 2.000 | 187.000 | 3.683 | 0.027 |
| condition | 0.004 | 0.001 | 3.000 | 187.000 | 0.405 | 0.750 |
| strength | 0.040 | 0.040 | 1.000 | 17.000 | 11.990 | 0.003 |
| time:condition | 0.008 | 0.001 | 6.000 | 187.000 | 0.402 | 0.877 |
| time:strength | 0.007 | 0.004 | 2.000 | 187.000 | 1.048 | 0.353 |
| condition:strength | 0.017 | 0.006 | 3.000 | 187.000 | 1.666 | 0.176 |
| time:condition:strength | 0.014 | 0.002 | 6.000 | 187.000 | 0.673 | 0.672 |
